# Impact of Supranormal LVEF After TAVI: Behavior, Mortality, and Cardiac Structure

**DOI:** 10.3390/jcm15072700

**Published:** 2026-04-02

**Authors:** Ximena Solar Sigala, Edgar Martínez, Carmen Olmos Blanco, Eduardo Pozo, Pedro Marcos-Alberca, José Juan Gómez de Diego, Patricia Mahía, Luis Nombela-Franco, Pilar Jiménez Quevedo, Gabriela Tirado, Luis Collado Yurrita, Maria Luaces, Antonio Fernández-Ortiz, Julián Pérez-Villacastín

**Affiliations:** 1 Cardiovascular Institute, Hospital Clínico San Carlos, 28040 Madrid, Spain; 2 Department of Medicine, Complutense University of Madrid, 28040 Madrid, Spain

**Keywords:** supranormal ejection fraction, aortic stenosis, concentric hypertrophy, transcatheter aortic valve implantation (TAVI)

## Abstract

**Background/Objectives:** Left ventricular ejection fraction (LVEF) typically improves after transcatheter aortic valve implantation (TAVI) in patients with severe aortic stenosis (SAS). However, the clinical significance and prognosis of patients presenting with supranormal LVEF (≥65%) remain poorly defined. This study aims to describe LVEF behavior, its relationship with mortality, and its effect on cardiac structure in this specific subgroup. **Methods:** A retrospective observational study was conducted at Hospital Clínico San Carlos (2008–2019), including SAS patients with pre-procedural supranormal LVEF. Patients were classified into two groups: those whose LVEF normalized (55–65%) and those whose LVEF remained supranormal. Demographic, clinical, and echocardiographic variables were collected at baseline and one-year follow-up. The primary endpoint was all-cause mortality at two years. **Results:** Out of 101 analyzed patients (mean age 82.8 years, 71.2% women), 71 (70.3%) experienced LVEF normalization at one year. Two-year mortality was 10% in the normalized group and 9.8% in the non-variable group, showing no significant difference. Regarding geometric characteristics, a trend toward left ventricular mass regression was observed only in the LVEF-normalized group (Delta −10; *p* = 0.062 vs. −8.4; *p* = 0.197). History of bleeding was the only variable showing a trend toward worse prognosis (*p* = 0.064). **Conclusions:** LVEF behavior one year after TAVI in patients with baseline supranormal function tends toward normalization. This change is not associated with differences in two-year mortality but is linked to a trend toward beneficial reverse cardiac remodeling.

## 1. Introduction

Aortic stenosis (AS) represents the most prevalent primary valvular heart disease requiring intervention in developed nations [1]. Driven by an aging global population, the burden of severe aortic stenosis (SAS) is projected to increase significantly over the coming decades. For years, surgical aortic valve replacement (SAVR) was the gold standard; however, the emergence and rapid evolution of transcatheter aortic valve implantation (TAVI) have revolutionized the therapeutic landscape. Initially reserved for inoperable or high-risk patients, TAVI is now routinely performed in intermediate and even low-risk cohorts, demonstrating non-inferior—and in many cases, superior—outcomes compared to traditional surgery [2,3,4].

The prognosis of patients undergoing TAVI is multifaceted. While technical success is high, long-term survival is heavily influenced by baseline cardiac function and the presence of comorbidities. Mortality rates post-TAVI vary widely, with reports ranging from 0.93% to 12.6% depending on age, frailty, and pre-existing myocardial damage [5,6]. Traditionally, clinical focus has been directed toward the assessment of the Left Ventricular Ejection Fraction (LVEF). It is well-established that patients with reduced LVEF (low-flow, low-gradient AS) face a higher risk of peri-procedural complications and all-cause mortality, as their myocardium may have already crossed the threshold into irreversible fibrosis [7,8].

In patients with baseline systolic dysfunction, the successful relief of pressure overload through TAVI often leads to significant LVEF recovery—a phenomenon known as “reverse remodeling.” This recovery is a powerful predictor of improved long-term survival [9,10]. However, our understanding of the opposite end of the spectrum remains surprisingly sparse. While the medical community has focused on the “failing” heart, data regarding LVEF changes in patients with supranormal function (LVEF ≥ 65–70%) remain limited and under-analyzed [11].

In 2019, heart failure with supranormal left ventricular ejection fraction (HFsnEF) was recognized as a distinct phenotypic entity [6,12]. This condition typically involves older patients, frequently women, with a history of hypertension and stiffened ventricles. Emerging evidence in general cardiology suggests a “U-shaped” mortality curve: while risk clearly increases when LVEF falls below 40%, there is a paradoxical increase in cardiovascular and non-cardiovascular mortality when LVEF exceeds 65–70% [13,14]. This “supranormal” state may not represent superior health, but rather a compensatory mechanism for a small, hypertrophied, and non-compliant ventricle.

Despite these insights in the general population, there is a profound lack of specific studies examining the relationship between supranormal LVEF and outcomes in the specific context of TAVI. After the relief of the valvular obstruction, the left ventricle undergoes an acute change in afterload. In some patients, this leads to “normalization” of the ejection fraction, while in others, the supranormal state persists. Whether this normalization represents a beneficial adaptation or a loss of compensatory reserve is currently unknown [15,16].

This study aims to address this gap in the literature by describing the behavior of LVEF in patients with baseline supranormal function undergoing TAVI at the Hospital Clínico San Carlos (Madrid, Spain). We aim to analyze the correlation between post-procedure LVEF changes and two-year mortality, as well as the impact on cardiac structure. Specifically, we assess changes in left ventricular geometry, including left ventricular mass index (LVMI) and relative wall thickness (RWT), to determine if LVEF normalization triggers a trend toward beneficial reverse remodeling and if it impacts long-term survival.

## 2. Materials and Methods

### 2.1. Study Design and Population

This is a retrospective, single-center observational study conducted at Hospital Clínico San Carlos (Madrid, Spain). We screened all patients diagnosed with severe aortic stenosis (SAS) who underwent transcatheter aortic valve implantation (TAVI) between January 2008 and December 2019. The inclusion criteria were: (1) patients aged > 18 years; (2) symptomatic SAS defined by an aortic valve area < 1 cm^2^ or index area < 0.6 cm^2^/m^2^; and (3) baseline supranormal left ventricular ejection fraction (LVEF), defined as LVEF > 65% measured by echocardiography. Exclusion criteria included: (1) unsuccessful procedure or periprocedural death; (2) prior surgical heart valve replacement; (3) significant primary mitral or tricuspid disease; and (4) loss to follow-up or death within the first 6 months (to ensure data for the one-year remodeling analysis).

### 2.2. Procedures and Follow-Up

TAVI was performed via transfemoral access in most cases using either balloon-expandable or self-expanding prostheses, according to the Heart Team’s consensus. Clinical follow-up was performed at 30 days, 6 months, 1 year, and annually thereafter. Demographic data, comorbidities (hypertension, diabetes, coronary artery disease), and risk scores (EuroSCORE II) were collected from electronic medical records.

### 2.3. Echocardiographic Assessment

Transthoracic echocardiography (TTE) was performed at baseline and at the one-year follow-up using standard equipment, GE Vivid (Chicago, IL, USA) or Philips EPIC (Amsterdam, The Netherlands). LVEF was calculated using the biplane Simpson’s method or the linear Teichholz method in cases of optimal endocardial border visualization [1,9]. Supranormal LVEF was defined as >65%, while normalization was defined as an LVEF between 55% and 65% according to international guidelines. Left ventricular geometry was assessed as follows: Left Ventricular Mass Index (LVMI): Calculated using the Devereux formula. Relative Wall Thickness (RWT): Calculated as (2 × posterior wall thickness)/end-diastolic diameter. Remodeling Patterns: Classified as concentric hypertrophy (RWT > 0.42 and increased LVMI) or eccentric hypertrophy based on standard ASE/EACVI criteria [1,13].

### 2.4. Endpoints and Definitions

The primary endpoint was all-cause mortality at two years (24 months) following the TAVI procedure. The secondary endpoint was the degree of structural reverse remodeling, specifically changes in LVMI and RWT between baseline and the one-year follow-up.

### 2.5. Statistical Analysis

Continuous variables are presented as mean standard deviation (SD) or median (interquartile range) as appropriate. Categorical variables are expressed as frequencies and percentages. Comparisons between groups (Persistent Supranormal vs. Normalized LVEF) were performed using Student’s *t*-test or Mann–Whitney U test for continuous variables and the Chi-square or Fisher’s exact test for categorical data. Survival curves were estimated using the Kaplan–Meier method and compared using the log-rank test. A Cox proportional hazards model was used to identify predictors of mortality. A *p*-value < 0.05 was considered statistically significant. All analyses were performed using Stata (College Station, TX, USA) version 17.0.

To account for early periprocedural mortality and to evaluate the prognostic impact of supranormal LVEF once the initial myocardial adaptation and structural remodeling had stabilized, a landmark analysis was performed. The landmark point was set at 6 months post-procedure. Consequently, only patients who survived at least 6 months after TAVI were included in the long-term survival analysis (24-month all-cause mortality), using Kaplan–Meier curves and Cox proportional hazards models. This approach ensures that the assessment of late clinical outcomes associated with post-TAVI LVEF categories is not confounded by early procedural complications or acute mortality.

During the preparation of this manuscript/study, the author(s) used Google Gemini AI tool for the purposes of reviewing English grammar. The authors have reviewed and edited the output and take full responsibility for the content of this publication.

## 3. Results

### 3.1. Patient Selection and Baseline Characteristics

A total of 138 patients with severe aortic stenosis (SAS) and baseline supranormal LVEF (≥65%) were initially screened. To ensure the evaluation of long-term myocardial behavior and mortality beyond the periprocedural period, we applied the 6-month landmark criteria.

**Figure 1:**
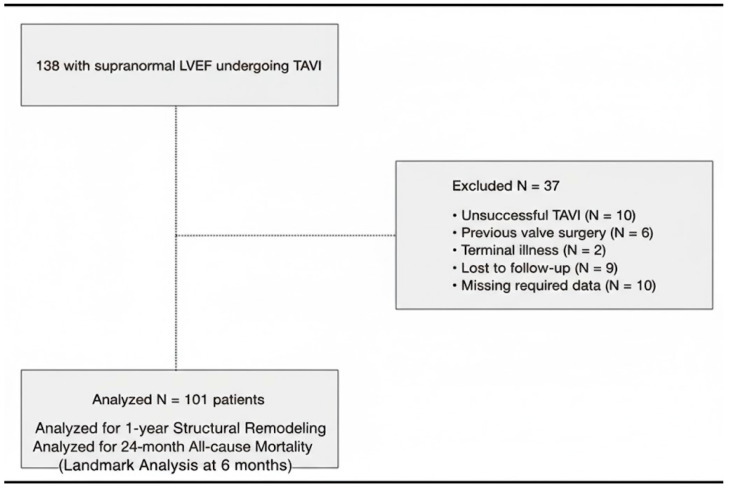
Flowchart of patient selection and study design. From an initial screening of 138 patients with severe aortic stenosis and supranormal LVEF, 101 met the inclusion criteria for the final analysis after excluding cases of unsuccessful procedures, prior surgeries, terminal illness, or incomplete follow-up. LVEF: Left Ventricular Ejection Fraction.

As shown in the study flowchart (Figure 1), 37 patients were excluded due to unsuccessful procedures (n = 10), previous valve surgeries (n = 6), terminal illnesses (n = 2), loss to follow-up (n = 9), and missing required data (n = 10). Consequently, a final cohort of 101 patients who survived at least 6 months post-TAVI was included for the 1-year structural remodeling and 24-month mortality analysis.

The mean age of the cohort was 82 ± 5 years, and 71.3% (n = 72) were women. Hypertension was the most prevalent comorbidity (82.2%), followed by dyslipidemia (58.4%) and atrial fibrillation (39.6%). No significant clinical differences were found between the groups, although patients in the non-variable LVEF group were slightly younger (81.1 vs. 83.4 years; *p* = 0.036). Baseline clinical data are summarized in Table 1.

### 3.2. Echocardiographic Evolution

**Figure 2:**
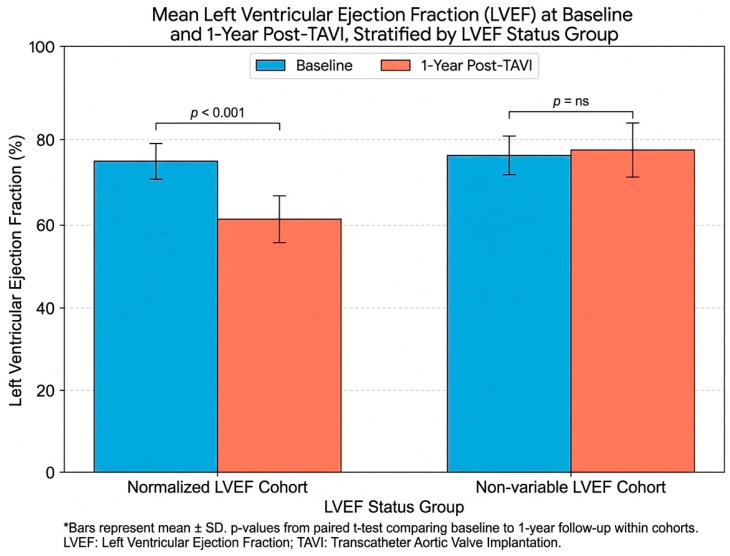
Comparison of LVEF at baseline and 1-year follow-up. In the Normalization group, a significant decrease from supranormal to physiological LVEF values is observed (*p* < 0.001), reflecting a relief of chronic pressure overload. The non-variable group maintained supranormal LVEF values throughout the study period. Data are presented as mean ± SD.

The baseline mean LVEF was 75.32% (SD 4.42). At the one-year follow-up, LVEF normalized in 70.3% of patients (n = 71) to a mean value of 61.17%, while it remained supranormal in 29.7% (n = 30) with a mean of 77.37% (*p* < 0.001), as shown in Figure 2.

Regarding structural changes, the baseline mean LV mass index (LVMI) was 123.70 g/m^2^ and the mean relative wall thickness (RWT) was 0.64. A trend toward reverse remodeling was observed specifically in the LVEF-normalized group, with a mean LV mass regression of −10 g/m^2^ (*p* = 0.062), compared to −8.4 g/m^2^ (*p* = 0.197) in the non-variable group. All echocardiographic parameters are detailed in Table 2.

### 3.3. Mortality Analysis

**Figure 3:**
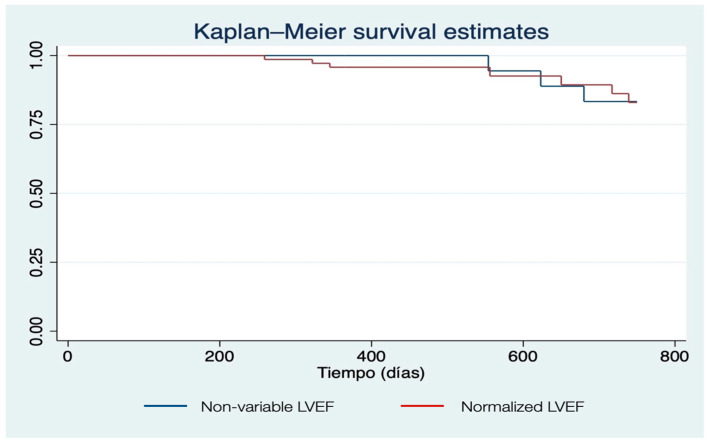
Kaplan–Meier survival curves comparing patients with LVEF normalization versus non-variable LVEF over a 24-month follow-up period (*p* > 0.05).

The overall all-cause mortality rate at the 24-month follow-up was 10% (n = 10). There was no statistically significant difference in mortality between the normalized LVEF group (10%) and the non-variable LVEF group (9.8%) [2,3]. Figure 3 displays the Kaplan–Meier survival estimates for both groups.

In the Cox proportional hazards regression analysis, a history of bleeding showed a trend toward an increased risk of mortality (HR 3.610; 95% CI 0.930–14.019; *p* = 0.064). Conversely, a higher baseline LVMI showed a non-significant trend toward lower mortality (HR 0.979; 95% CI 0.958–1.001; *p* = 0.067). Predictors of two-year mortality are presented in Table 3.

## 4. Discussion

The present study explores the clinical and echocardiographic implications of supranormal LVEF in elderly patients undergoing TAVI. Our main finding is that “normalization” of LVEF (to ranges between 55 and 65%) is a frequent phenomenon (70.3%) that reflects a positive adaptation to the relief of pressure overload, rather than myocardial deterioration.

### 4.1. The Hemodynamic Basis of LVEF Normalization

In the setting of severe and chronic aortic stenosis, the presence of a supranormal LVEF often represents a complex compensatory hyperdynamic state. This physiological response is triggered by the necessity to maintain an adequate cardiac output and stroke volume against a persistently high and debilitating afterload [13]. Our findings demonstrate that once the transcatheter aortic valve implantation (TAVI) procedure effectively relieves this mechanical obstruction, the LVEF tends to transition back toward standard physiological ranges (decreasing from a baseline mean of 75% to 61% at follow-up, *p* < 0.001).

This phenomenon of ‘normalization’ should be interpreted as a direct and expected hemodynamic consequence of the sudden and significant reduction in ventricular wall stress and myocardial oxygen demand following the valve replacement [14]. Crucially, as demonstrated in our clinical cohort, this numerical drop in LVEF does not translate into a deterioration of cardiac function or worse clinical outcomes. On the contrary, it reflects a stabilization of the left ventricle’s workload, as evidenced by the similar and stable 24-month survival rates observed between the study groups (Figure 3). This suggests that the ‘supranormal’ state was a marker of excessive compensatory effort that is no longer required once the transcatheter bioprosthesis is in place.

### 4.2. Age and Myocardial Response

A distinctive finding was that older patients were more likely to normalize their LVEF (83.47 vs. 81.13 years; *p* = 0.036). This could be explained by a higher degree of myocardial senescence and interstitial fibrosis in older individuals, which may limit the heart’s ability to sustain a hyperdynamic state once the trigger (the stenosis) is removed [15,16]. Furthermore, the lack of LVEF normalization in some patients could be associated with more advanced stages of myocardial fibrosis or a longer duration of chronic pressure overload prior to TAVI. In these cases, irreversible structural damage may limit the capacity for immediate reverse remodeling, even after the successful relief of valvular obstruction.

### 4.3. Reverse Structural Remodeling

We observed a relevant trend toward greater regression of LV mass in the normalization group (10 g/m^2^; *p* = 0.062) compared to the non-variable group. This suggests that the reduction in LVEF to normal ranges may be a marker of more effective reverse remodeling. The high baseline RWT (0.64) in our population indicates a state of extreme concentric hypertrophy that requires this “relaxation” of the ejection fraction to initiate structural recovery [17].

### 4.4. Predictors of Mortality

Finally, our analysis (Table 3) confirmed that in this specific population with supranormal LVEF, traditional risk factors do not predict 2-year mortality. Only a history of prior bleeding showed a trend toward significance (*p* = 0.064), which is consistent with recent registries suggesting that non-cardiac comorbidities often dictate the prognosis in the very elderly TAVI population. However, it must be acknowledged that our study is underpowered to detect small differences in mortality due to the relatively small sample size (n = 101) and the low event rate. This inherent limitation increases the risk of a Type II error; therefore, the lack of statistical significance in mortality comparisons should be interpreted with caution and viewed as hypothesis-generating.

### 4.5. Limitations

This study has several limitations. First, its retrospective and single-center nature may limit the generalizability of the results. Second, the sample size (n = 101) might be underpowered to detect small differences in mortality between subgroups. Third, LVEF was primarily measured using Simpson’s biplane method; however, in cases with suboptimal acoustic windows, Teichholz or visual estimation was used. This introduces a potential risk of patient misclassification, particularly near the study’s established thresholds.

Furthermore, the study is underpowered to detect small differences in mortality due to the relatively small sample size and the low event rate. This inherent limitation increases the risk of a Type II error; therefore, the lack of statistical significance in mortality comparisons should be viewed with caution and not as definitive proof of equivalence. Despite these constraints, our data provide a valuable preliminary signal regarding myocardial adaptation and structural recovery that warrants further investigation.

## 5. Conclusions

In summary, the findings of the present study provide a novel perspective on the clinical and functional trajectory of elderly patients undergoing TAVI who exhibit a supranormal left ventricular ejection fraction at baseline. Our data suggests that the phenomenon of LVEF normalization—defined as the transition of the ejection fraction toward the standard physiological range of 55–65%—is an exceedingly frequent and physiologically appropriate adaptation following the successful relief of chronic and severe pressure overload. This functional shift should not be misinterpreted by clinicians as a sign of myocardial failure or deterioration; rather, it represents a favorable hemodynamic stabilization and a necessary reduction in the hyperdynamic compensatory state that the heart is forced to maintain to overcome valvular obstruction.

Furthermore, while the process of LVEF normalization was not associated with a statistically significant reduction in 2-year all-cause mortality within our specific cohort, it appears to serve as a key clinical trigger for a beneficial trend toward reverse structural remodeling. The observed regression in left ventricular mass and the favorable changes in myocardial geometry suggest that these patients are moving toward a more efficient cardiac state.

Nevertheless, it is imperative to acknowledge the inherent statistical limitations of this analysis. Given the relatively small sample size and the low event rate, our results must be strictly interpreted as hypothesis-generating. The potential risk of a Type II error necessitates that the lack of prognostic significance in mortality be viewed with caution. Therefore, further large-scale, prospective, and multicenter investigations are warranted to definitively determine the long-term prognostic value of different LVEF trajectories. Ultimately, these insights contribute to a more nuanced understanding of myocardial recovery in the increasingly complex population of patients treated with transcatheter valve therapies.

## Figures and Tables

**Table 1:** Baseline clinical characteristics of patients who normalized LVEF and those in whom no significant variation occurred.

Clinical Characteristics	All Patients (n = 101)	Normalized LVEF (n = 71)	Non-Variable LVEF (n = 30)	*p* Value
**Women, n (%)**	72 (71.28%)	50 (70.42%)	22 (73.3%)	0.768
**Age (years, mean ± SD)**	82.81 ± 5.14	83.47 ± 5.39	81.13 ± 4.19	0.036
**Hypertension, n (%)**	84 (82.17%)	57 (80.28%)	27 (83.33%)	0.444
**DLP, n (%)**	59 (58.41%)	40 (56.33%)	19 (63.33%)	0.515
**Diabetes, n (%)**	31 (30.69%)	19 (26.7%)	12 (40%)	0.187
**CC, n (%)**	14 (13.86%)	10 (14.08%)	4 (13.33%)	0.928
**Tobacco, n (%)**	15 (14.85%)	10 (14.08%)	5 (16.66%)	0.739
**History of bleeding, n (%)**	9 (8.91%)	4 (5.63%)	5 (16.66%)	0.075
**Creatinine (mg/dL)**	1.07 ± 0.48	1.12 ± 0.51	0.96 ± 0.39	0.130
**eGFR MDRD (mL/min/1.73 m^2^)**	63.44 ± 23.81	61.12 ± 24.77	69.72 ± 21.14	0.127
**Stroke, n (%)**	14 (13.86%)	11 (15.49%)	3 (10%)	0.465
**Cancer in remission, n (%)**	12 (11.88%)	9 (8.91%)	3 (10%)	0.704
**AF, n (%)**	40 (39.6%)	25 (24.75%)	15 (50%)	0.165
**log_Euroscore**	13.87 ± 5.61	13.73 ± 6.78	14.84 ± 8.10	0.588
**Euroscore II**	4.13 ± 1.84	4.23 ± 1.84	3.83 ± 2.89	0.709

Data are expressed as n (%) or mean ± SD. DLP: Dyslipidemia; CC: Chronic Complications; eGFR MDRD: estimated Glomerular Filtration Rate; AF: Atrial Fibrillation.

**Table 2:** Echocardiographic characteristics at baseline and at 1 year post-TAVI.

Clinical Characteristics	All Patients (n = 101)	Normalized LVEF (n = 71)	Non-Variable LVEF (n = 30)	*p*-Value
**LVEF (%)**	75.32 ± 4.42	74.73 ± 4.18	76.10 ± 4.56	0.077
**LVEDD (mm)**	43.74 ± 6.97	43.83 ± 7.36	43.65 ± 6.31	0.888
**IVS (mm)**	14.25 ± 2.70	14.16 ± 2.54	14.45 ± 3.13	0.749
**PW (mm)**	12.50 ± 1.94	12.40 ± 2.00	12.73 ± 1.84	0.629
**Indexed diastolic volume (mL/m^2^)**	44.39 ± 14.23	44.89 ± 15.15	43.17 ± 12.20	0.466
**LVMI (g/m^2^)**	123.70 ± 30.41	122.74 ± 30.47	126.39 ± 31.17	0.589
**RWT**	0.64 ± 0.15	0.64 ± 0.16	0.65 ± 0.13	0.628
**LVOT systolic volume (mL/m^2^)**	39.84 ± 11.74	40.12 ± 11.87	43.17 ± 12.20	0.706
**AVA (cm^2^)**	0.64 ± 0.18	0.64 ± 0.18	0.64 ± 0.18	0.896
**Peak Ao gradient (mmHg)**	82.30 ± 20.49	81.64 ± 16.86	84.96 ± 27.71	0.628
**Mean Ao gradient (mmHg)**	49.56 ± 12.75	50.17 ± 11.9	48.60 ± 14.87	0.498
**LVEF (%) Post-TAVI**	66.08 ± 7.40	61.17 ± 5.40	77.37 ± 6.32	0.000

LVEF: Left Ventricular Ejection Fraction; LVEDD: Left Ventricular End-Diastolic Diameter; IVS: Interventricular Septum; PW: Posterior Wall; LVMI: Left Ventricular Mass Index; RWT: Relative Wall Thickness; LVOT: Left Ventricular Outflow Tract; AVA: Aortic Valve Area.

**Table 3:** Univariate and Multivariable Cox Proportional Hazards Analysis for 24-month All-cause Mortality.

Variable	Univariate HR (95% CI)	*p*-Value	Multivariable HR (95% CI) *	*p*-Value
**LVEF Normalization**	1.02 (0.22–4.75)	0.980	1.05 (0.21–5.12)	0.950
**Female Sex**	0.870 (0.224–3.367)	0.840	-	-
**Age (per year)**	1.017 (0.901–1.148)	0.776	-	-
**Body Mass Index**	0.932 (0.800–1.086)	0.372	-	-
**Hypertension**	1.371 (0.172–10.902)	0.765	-	-
**Dyslipidemia**	0.728 (0.210–2.515)	0.616	-	-
**Diabetes Mellitus**	0.499 (0.106–2.325)	0.381	-	-
**Coronary Artery Disease**	0.825 (0.104–6.548)	0.856	-	-
**Smoking**	1.708 (0.480–4.292)	0.254	-	-
**History of bleeding**	3.610 (0.930–14.019)	0.064	-	-
**Creatinine**	1.333 (0.413–4.308)	0.630	-	-
**Stroke**	1.589 (0.320–7.112)	0.603	-	-
**Atrial Fibrillation**	1.077 (0.309–3.757)	0.309	-	-
**EuroSCORE II**	0.797 (0.539–1.178)	0.256	-	-

* Note: To prevent statistical overfitting given the limited number of events (n = 10 deaths), the multivariable Cox model was restricted to the primary study variable (LVEF Normalization) to adhere to the 10 events per variable (EPV) principle.

## Data Availability

The data presented in this study are available upon request from the corresponding author due to privacy and ethical restrictions.

## Abbreviations

SAS	Severe Aortic Stenosis
LV	Left Ventricle
TAVI	Transcatheter Aortic Valve Implantation
LVEF	Left Ventricular Ejection Fraction
RVDD	Right Ventricular Diastolic Diameter
IVS	Interventricular Septum thickness
LVPW	Left Ventricular Posterior Wall thickness
LVMI	Left Ventricular Mass Index
RWT	Relative Wall Thickness
LVOT	Left Ventricular Outflow Tract
AVA	Aortic Valve Area
HTN	Hypertension
DLP	Dyslipidemia
CAD	Coronary Artery Disease
Cr	Creatinine
GFR	Glomerular Filtration Rate
CVA	(Cerebrovascular Accident)/Stroke
AF	Atrial Fibrillation
HR	Hazard Ratio
